# Clinical Significance of Serum Elafin in Children with Inflammatory Bowel Disease

**DOI:** 10.3390/biomedicines10123267

**Published:** 2022-12-16

**Authors:** Paulina Krawiec, Elżbieta Pac-Kożuchowska

**Affiliations:** Department of Pediatrics and Gastroenterology, Medical University of Lublin, Al. Racławickie 1, 20-059 Lublin, Poland

**Keywords:** Crohn’s disease, ulcerative colitis, host defense peptide, antiproteinase

## Abstract

Background: The role of elafin in the pathophysiology of inflammatory bowel disease (IBD) has not been not elucidated. We aimed to evaluate serum elafin in children with IBD and assess its relationship with disease activity. Methods: We enrolled children with IBD in the study group and children with functional abdominal pain in the control group. We evaluated serum elafin using enzyme-linked immunosorbent assay kits. Results: In children with IBD, serum elafin (mean ± SD: 4.192 ± 1.424 ng/mL) was significantly elevated compared with controls (mean ± SD: 3.029 ± 1.366 ng/mL) (*p* = 0.0005). Elafin was significantly increased in children in the active phase of IBD (mean ± SD: 4.424 ± 1.449 ng/mL) compared with the control group (*p* = 0.0003). In IBD remission, only children with ulcerative colitis (mean ± SD: 4.054 ± 1.536 ng/mL) had elevated elafin compared with controls (*p* = 0.004). ROC analysis revealed that the area under the curve (AUC) of serum elafin was 0.809 while discriminating patients with ulcerative colitis from the control group, and the AUC was 0.664 while differentiating patients with Crohn’s disease from the control group. Conclusions: Serum elafin was found to be elevated in our cohort of children with IBD, depending on disease activity. Serum elafin was increased in the active phases of both ulcerative colitis and Crohn’s disease, but only in the remission of ulcerative colitis. Elafin appears to be a potential candidate for a biomarker of ulcerative colitis.

## 1. Introduction

Inflammatory bowel disease (IBD) refers to a group of lifelong inflammatory disorders of the gastrointestinal tract, including Crohn’s disease and ulcerative colitis, which are characterized by an unpredictable relapsing and remitting course [1]. Although IBD is usually diagnosed in young adulthood, it may affect all age groups, from infants to the elderly [2]. The incidence of IBD has been increasing in pediatric and adult populations worldwide [1,3]. In general, pediatric-onset IBD appears to be associated with a more severe course, complicated disease behavior, and more frequent disease extension [2]. In addition to its significant impact on physical health, IBD may affect psychosocial aspects of life and decrease health-related quality of life [4,5]. IBD in children and adolescents may be associated with difficulties in behavioral and emotional functioning, unfavorable implications in education, and disruption in family and school functioning [4,5]. The increase in IBD prevalence and myriad of negative health outcomes challenge scientists to improve the diagnostics and therapy of pediatric IBD [6].

Many research studies have focused on the mechanisms that underlie the development of IBD. A thorough understanding of IBD pathophysiology may guide scientists to identify novel therapeutic target points and establish markers useful in the diagnosis and monitoring of the IBD course [7]. Although considerable progress has recently been made in our understanding of the pathogenesis of IBD, little is known about the role of host defense peptides (HDPs) in chronic gastrointestinal inflammation. Host defense peptides exhibit antimicrobial activity and display complex immunomodulatory functions, exerting both pro- and anti-inflammatory properties depending on their expression level at the site of inflammation [8,9,10].

Elafin is one of the HDPs that exhibits antimicrobial and antiprotease activity [11]. Elafin, also called skin-derived antileuko protease or peptidase inhibitor-3, is a cationic, 95-amino-acid protein of low molecular weight (6 kDa). The protein consists of two domains. The globular C-terminal end presents folding and four disulphide bonds, and it exhibits protease inhibition activity. The flexible NH2 domain is a substrate for transglutaminase, which allows the formation of elafin polymers or the binding of elafin to extracellular matrix proteins [12,13,14]. 

Initially, elafin was isolated from psoriatic skin [15]. However, it is also synthesized by epithelial cells of the gastrointestinal tract, lungs, or female reproductive system and inflammatory cells, including neutrophils, mast cells, and macrophages [11,12].

Elafin is an alarm antiproteinase that inhibits the neutrophil-derived serine proteases elastase and proteinase-3 through a competitive tight-binding mechanism, protecting tissues against elastolytic destruction [12,13]. In addition to its antiprotease properties, it was revealed that elafin may exhibit pleiotropic effects including antimicrobial activity, suppression of inflammation, immunoregulation, involvement in tissue remodeling, and cell differentiation [13].

Until now, there have been some studies regarding the role of elafin in the pathophysiology of different diseases, including psoriasis, pulmonary hypertension, and systemic sclerosis [16,17,18]. However, there have been only a few studies focusing on elafin in inflammatory bowel disease that delivered conflicting results [19,20,21,22,23]. Thus, this study aimed to evaluate the serum concentration of elafin in children with IBD in relation to the phenotype and clinical activity of the disease and inflammatory markers.

## 2. Materials and Methods

### 2.1. Study and Control Group

For the study group, we enrolled patients aged ≤ 18 years with IBD who were hospitalized at the Department of Pediatrics and Gastroenterology, Medical University of Lublin, Poland, between June 2017 and October 2019 [24,25]. IBD was recognized based on a combination of clinical manifestations, laboratory and imaging studies, and gastrointestinal tract endoscopy with histology according to the revised Porto criteria [26]. There are two main subtypes of IBD, i.e., ulcerative colitis and Crohn’s disease. Typically, ulcerative colitis is characterized as a continuous inflammation of the mucosa starting from the rectum and extending for a variable distance of the colon, without small bowel involvement [26]. The presence of crypt architectural distortion and basal plasmacytosis and the lack of epithelioid granulomas in the biopsy support the diagnosis of ulcerative colitis [26]. Typical Crohn’s disease can be described as a noncontinuous, granulomatous, and transmural inflammatory process that may involve any gastrointestinal tract region [26].

We excluded children with IBD who had any clinical or laboratory signs of acute infection at the time of enrolment and/or a history of surgery within the 4 weeks prior to enrolment. Another exclusion criterion was the lack of informed consent of parents and/or patients aged ≥16 years old [24,25]. In all IBD children, we evaluated the disease phenotype with the use of the Paris Classification [27]. Patients were divided into two groups according to the clinical activity of the disease, i.e., with active IBD and in IBD remission. Disease activity was established based on the Pediatric Crohn’s Disease Activity Index (PCDAI) [28] and the Pediatric Ulcerative Colitis Activity Index (PUCAI) in patients with Crohn’s disease and ulcerative colitis, respectively [29]. Clinical remission of Crohn’s disease was defined as a PCDAI score ≤ 10 points [28], while clinical remission of ulcerative colitis was defined as a PUCAI score < 10 points [29].

For the control group, we enrolled children with functional abdominal pain recognized based on the Rome IV Diagnostic Criteria for Functional Gastrointestinal Disorders [30]. We excluded children with a history of any concomitant organic disease, a surgery within the 4 weeks prior the enrolment, or any clinical or laboratory signs of acute or chronic inflammation at the time of recruitment. Another exclusion criterion was the lack of informed consent of parents and/or patients aged ≥16 years old [24,25]. 

We enrolled 68 children with IBD in the study group. There were 43 patients with ulcerative colitis and 25 with Crohn’s disease. The age of patients with ulcerative colitis ranged from 6.5 to 18 years old (median: 14.5 years old; mean: 13.6 ± 3.4 years old), while patients with Crohn’s disease ranged from 8.5 to 18 years old (median: 13.5 years old; mean: 13.7 ± 2.6 years old).

Most patients had clinically active IBD (39; 57.4%), including 23 patients with ulcerative colitis and 16 with Crohn’s disease, and received IBD-specific medications (41; 60.3%). The baseline characteristics of patients with IBD, including disease location, clinical activity, and medication use, have been published previously [24,25].

The control group consisted of 20 children with functional abdominal pain. There was a slight girl preponderance (12; 60%) in the control group. The mean age of children was 11.9 ± 3.47 years old (median: 12.25 years old; range: 4.5–17.5 years old) [24,25].

### 2.2. Methods

Peripheral blood samples were obtained from children with IBD and controls to determine the complete blood count (CBC), C-reactive protein (CRP), erythrocyte sedimentation rate (ESR), and elafin level. The CBC, CRP, and ESR were determined according to standard laboratory practice. Serum elafin was measured using commercially available enzyme-linked immunosorbent assay kits (ELISA assay kit for peptidase inhibitor-3, skin-derived PI3; human) according to the manufacturer’s recommendations (Cloud-Clone Corp., Katy, TX, USA, Serial No. 723997EFFB). Stool samples were obtained from children with IBD to evaluate fecal calprotectin using quantitative chemiluminescent sandwich immunoassay (CLIA) according to the manufacturer’s recommendations (LIAISON Calprotectin assay Ref. 318960; DiaSorin Inc., Stillwater, MN, USA).

### 2.3. Statistical Analysis

The statistical analysis was performed using Statistica v. 13.3 software (StatSoft Polska Sp. z o.o., Kraków, Poland). The data are presented as mean and standard deviation, or median and range. To test differences between two groups, a Mann–Whitney U-rank test was applied. To test differences between more than two means for more than two groups, a H Kruskal–Wallis test was used. Correlations between parameters were analyzed using the Spearman’s rank correlation coefficient. Values of *p* < 0.05 were considered statistically significant.

We performed receiver operating characteristic (ROC) analysis to determine the discriminatory capacity of serum elafin in distinguishing patients with IBD from children in the control group. The area under the receiver operating curve (AUC), 95% confidence intervals (CI), and standard error (SE) were established. Diagnostic accuracy measures for elafin, including sensitivity, specificity, accuracy, positive predictive value, and negative predictive value, were calculated.

### 2.4. Ethical Approval and Funding

Written informed consent for participation in this study was obtained from a patient’s parent and from the patient in the case of a child aged ≥16 years. This study was approved by the Bioethical Committee of the Medical University of Lublin (KE-0254/289/2016).

This study was funded by the Medical University of Lublin (grant no. MNmb466) to PK. The article processing charge (APC) was funded by the Medical University of Lublin, grant no. DS406 to EP-K.

## 3. Results

**Figure 1:**
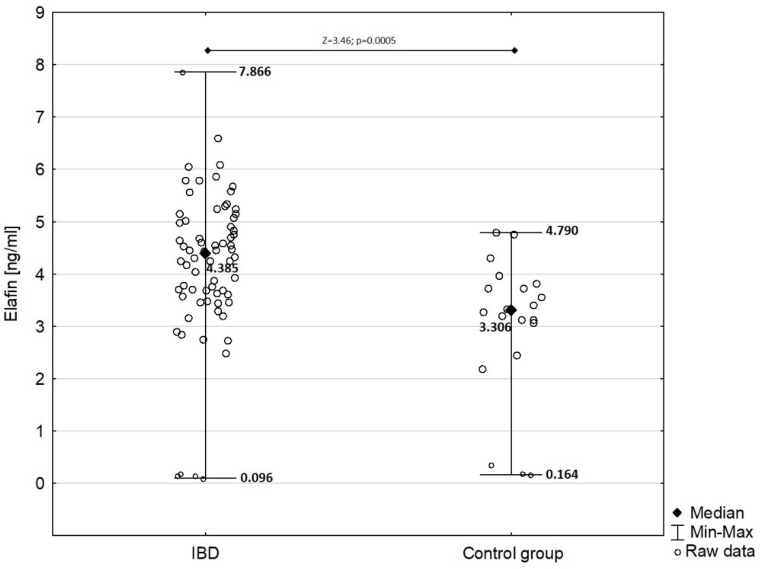
Comparison of serum elafin between children with IBD and children from control group.

In children with IBD, serum elafin was significantly elevated (mean ± SD: 4.192 ± 1.424 ng/mL) compared with that in the control group (mean ± SD: 3.029 ± 1.366 ng/mL) as presented in Figure 1 (Z = −3.46; *p* = 0.0005).

**Figure 2:**
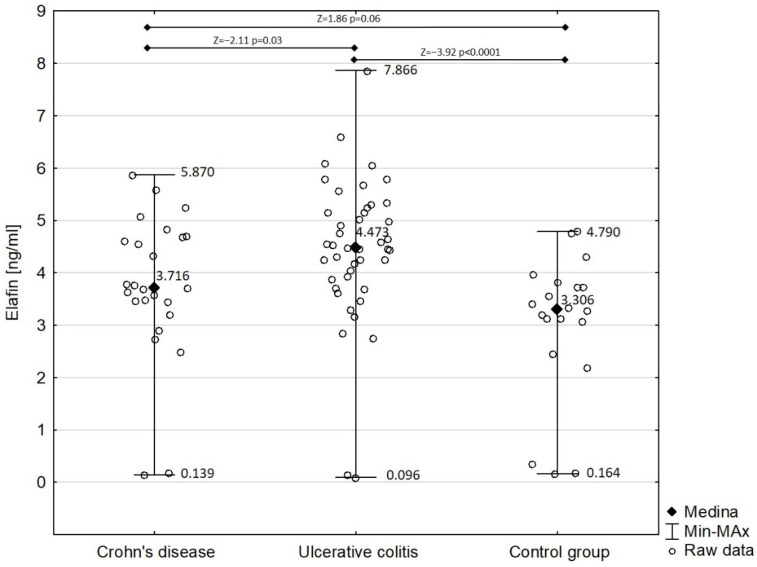
Serum elafin in patients with Crohn’s disease and ulcerative colitis in comparison with control group.

Figure 2 shows the comparison of serum elafin between children with Crohn’s disease and ulcerative colitis and the control group. Detailed analysis demonstrated that the concentration of serum elafin was significantly increased in ulcerative colitis patients (mean ± SD: 4.450 ± 1.395 ng/mL) compared with in the control group (Z = −3.92; *p* < 0.0001). However, the difference in serum elafin between Crohn’s disease patients (mean ± SD: 3.749 ± 1.390 ng/mL) and the control group almost attained significance (Z = 1.86; *p* = 0.06). Elafin was significantly higher in children with ulcerative colitis compared with those with Crohn’s disease (Z = −2.11; *p* = 0.03). The concentration of serum elafin in girls with IBD (4.067 ± 1.311 ng/mL) did not significantly differ from that in boys in the study group (4.324 ± 1.544 ng/mL) (Z = −0.83; *p* = 0.40).

There were significant differences in elafin concentration when comparing IBD patients in terms of clinical disease activity to the control group. Elafin was significantly increased in children in an active phase of IBD (mean ± SD: 4.424 ± 1.449 ng/mL; median: 4.537 ng/mL; range: 0.139–7.866 ng/mL) compared with the control group (Z = 3.66; *p* = 0.0003). We also found significant differences while comparing elafin in patients with IBD remission (mean ± SD: 3.881 ± 1.351 ng/mL; median: 3.935 ng/mL; range: 0.096–5.797 ng/mL) and controls (Z = −2.41; *p* = 0.02). Moreover, we performed a detailed analysis comparing the concentration of serum elafin in patients in an active phase and remission of IBD with that in healthy controls, which is presented in Table 1.

Compared with the control group, elafin was significantly increased in IBD patients, regardless of whether they were treatment-naïve (mean ± SD: 4.357 ± 1.247 ng/mL; median: 4.469 ng/mL; range: 0.139–6.601 ng/mL) or received any therapy (mean ± SD: 4.083 ± 1.535 ng/mL; median: 4.263 ng/mL; range: 0.096–7.866 ng/mL) (H = 12.66; *p* = 0.002). However, there were no significant differences in the concentration of elafin between treatment-naïve children and those who received any IBD therapy (Z = 0.75; *p* = 0.45).

Subgroup analysis did not reveal any significant differences in elafin concentration among IBD children depending on the disease location in both ulcerative colitis (H = 5.18; *p* = 0.27) and Crohn’s disease (H = 0.84; *p* = 0.83). 

In patients with Crohn’s disease, we found positive correlations between elafin and CRP (*p* = 0.01; R = 0.49) and fecal calprotectin (*p* = 0.03; R = 0.46). However, there was no significant correlation between serum elafin and PCDAI (*p* = 0.06; R = 0.38) and ESR (*p* = 0.25; R = 0.24). In patients with ulcerative colitis, there were no significant correlations between serum elafin and inflammatory markers, i.e., ESR (*p* = 0.41; R = 0.13), CRP (*p* = 0.38; R = 0.14), fecal calprotectin (*p* = 0.78; R = −0.05), and the clinical activity index PUCAI (*p* = 0.23; R = 0.19).

**Figure 3:**
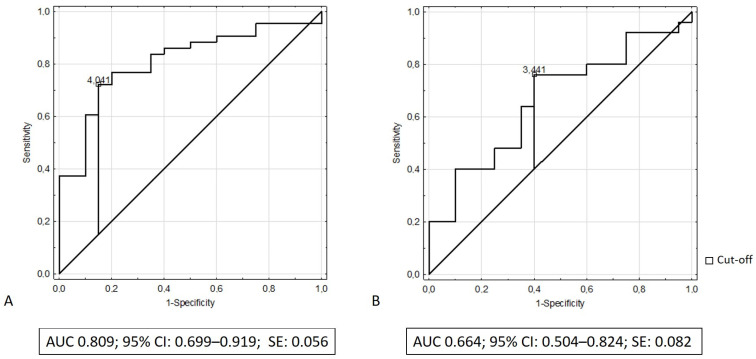
(**A**) ROC curve and area under ROC for elafin in recognition of ulcerative colitis; (**B**) ROC curve and area under ROC for elafin in recognition of Crohn’s disease.

We performed receiver operating characteristic (ROC) analysis to determine the discriminatory capacity of serum elafin in distinguishing patients with IBD from the control group. The results of ROC analysis revealed that the area under the curve (AUC) of serum elafin was 0.809 (95% CI: 0.699–0.919; SE: 0.056) while discriminating patients with ulcerative colitis from the control group and that the AUC was 0.664 while differentiating patients with Crohn’s disease from the control group (95% CI: 0.504–0.824; SE: 0.082). Figure 3A,B illustrate the receiving operating characteristic curves for elafin in the recognition of ulcerative colitis and Crohn’s disease, respectively. Table 2 presents the diagnostic performance of elafin for the recognition of ulcerative colitis and Crohn’s disease.

## 4. Discussion

To the best of our knowledge, this is one of the first studies assessing the concentration of serum elafin in children with IBD. We demonstrated that elafin was significantly elevated in patients with active ulcerative colitis and Crohn’s disease compared with in the control group. However, only patients in remission of ulcerative colitis but not Crohn’s disease had higher concentrations of serum elafin compared with controls. Elafin was also significantly higher in children with ulcerative colitis compared with those with Crohn’s disease. Moreover, treatment status did not affect elafin elevation in IBD children compared with controls.

Our results are in accordance with some previous findings [19,20,23]. In a recent study by Wang et al., serum elafin was significantly increased in patients with ulcerative colitis compared with the control group, while in Crohn’s disease patients there was a trend toward mildly elevated elafin, which was not statistically significant [23]. Moreover, colonic elafin mRNA expression and elafin protein expression were significantly elevated in patients with ulcerative colitis compared with in the control group [23]. However, elafin mRNA and protein expression did not significantly differ between patients with Crohn’s disease and controls [23]. Interestingly, patients with stricturing Crohn’s disease had higher mesenteric fat elafin mRNA expression than patients with nonstricturing Crohn’s disease [23].

Schmid et al. found that elafin mRNA was expressed predominantly in inflamed ulcerative colitis colonic biopsies [20]. Moreover, it was reporetd that elafin mRNA was significantly induced in inflamed versus noninflamed ulcerative colitis colonic biopsies and in inflamed versus noninflamed Crohn’s disease colonic biopsies [20]. However, there was a sixteen-fold increase in noninflamed versus inflamed ulcerative colitis biopsies compared with only a three-fold increase in noninflamed versus inflamed Crohn’s disease biopsies [20]. The difference between Crohn’s disease and ulcerative colitis was also statistically significant [20].

Furthermore, whole-genome microarray screenings revealed the upregulation of elafin gene transcripts in the active rectal mucosa from patients with ulcerative colitis compared with controls, suggesting that elafin may be a candidate biomarker of ulcerative colitis [19].

During inflammation, including IBD, the equilibrium between serine proteases and their endogenous inhibitors might be shifted toward proteases [31]. Aggravated activity of proteases in the gastrointestinal system may be involved in the initiation and propagation of inflammation through the degradation of extracellular matrix proteins, proteolytic activation of inflammatory mediators, activation of intracellular pathways of inflammation, induction of mucosal layer apoptosis, and inhibition of leukocyte apoptosis [12,31]. Thus, increased elafin in IBD may be considered a defense mechanism attenuating some stages of the inflammatory cascade [12,20]. It was shown in a trinitrobenzene sulfonic acid (TNBS) and dextran sodium sulfate (DSS)-induced colitis model that overexpression of elafin led to restoration of proteolytic balance; downregulation of IL-6, IL-8, IL-17A, and NFκΒ; and inhibition of TNF-α–induced increased permeability [32].

On the contrary, Motta et al. showed that mucosal expression of elafin was diminished in patients with IBD, which could result in increased elastolytic activity in colon tissue collected from these patients [21]. Furthermore, Zhang et al. found significantly decreased expression of elafin mRNA in peripheral blood leukocytes in patients with active ulcerative colitis and increased expression in ulcerative colitis remission. However, there were no significant differences in elafin mRNA between patients with Crohn’s disease and controls [22]. It was also demonstrated that the expression of elafin in inflamed colonic mucosa in IBD patients was significantly decreased compared with in noninflamed mucosa in IBD patients and in the colonic specimens from controls [22]. Zhang et al. assessed serum elafin, which was also decreased in both patients in an active phase and IBD remission [22]. Downregulation of elafin in IBD patients compared with controls may suggest that disruption of the protease/antiprotease balance may take part in IBD development [22]. Another explanation for decreased elafin expression in IBD is that it may be a consequence of chronic inflammation leading to the destruction of epithelial cells, which are the main source of elafin [22].

The differences between the serum level of elafin and its colonic expression inspired further experiments by Wang et al. to establish the source of elafin. It was observed that exposure to the serum exosomes from IBD patients decreased elafin expression in the peripheral blood mononuclear cells from healthy donors [23]. Moreover, Wang et al. found that the serum exosomes from patients with stricturing Crohn’s disease elevated the secretion of elafin. Thus, mesenteric fat adipocytes appear to be an important source of elafin in the circulation of patients with stricturing Crohn’s disease [23].

Inconsistent results for elafin in IBD patients may result from its dual role in inflammation. On the one hand, elafin as an alarm antiprotease may protect tissues against destruction by proteases during inflammation and exhibit anti-inflammatory activity through the inhibition of the recruitment and action of inflammatory cells [13]. It may inhibit the action of the transcription factor NF-κB and the release of proinflammatory cytokines such as TNF and IL-8 [33]. Moreover, it may protect against inflammation by promoting the resolution of the inflammatory cascade through inhibiting human neutrophil elastase-mediated cleavage of macrophages and restoring the capacity of macrophages to recognize apoptotic cells [13,34,35].

On the other hand, elafin may promote innate and adaptive immune responses [13]. It has been shown that elafin significantly augments LPS-mediated neutrophil migration and enhances TNF-α production in macrophages [36,37,38]. Moreover, results from Sallenave et al. suggested that elafin may concomitantly upregulate local (pulmonary) innate immunity and downregulate systemic inflammatory responses in the circulation [38]. It was also shown that elafin may exhibit the potential for modulating both humoral and cell-mediated adaptive immune responses [39]. Elafin elevated the number of dendritic cells and lymphocytes; favored the activation status of dendritic cells; and induced increased the expression of IL-12, IFN-γ, and local and systemic antibodies [13,39]. Although some insight into the function of elafin has been gained, the mechanism of its action in the regulation of immune response has not yet been established.

To date, there has been no research evaluating the diagnostic accuracy of elafin in IBD. Thus, we performed ROC analysis, which revealed a moderate discriminatory capacity of serum elafin in distinguishing children with ulcerative colitis from controls. However, further longitudinal studies are needed to fully determine elafin’s usefulness in the recognition and monitoring of IBD activity in children and adolescents. Although there was a report of some sex-specific differences in the IBD course and phenotype, we did not find significant differences in the concentration of serum elafin between girls and boys with IBD [40].

In our study, we found positive correlations between serum elafin and CRP and fecal calprotectin only in patients with Crohn’s disease. Zhang et al. found that elafin mRNA was negatively correlated with ESR, CRP, and the modified Mayo score in patients with ulcerative colitis and with the Best Crohn’s Disease Activity Index in patients with Crohn’s disease [22]. Wang et al. revealed positive correlations between serum elafin and the Harvey Bradshaw Index and the Partial Mayo Score [23]. However, there was no significant association between serum elafin and endoscopic IBD activity indices, i.e., the Simple Endoscopic Score for Crohn’s disease and the Mayo Endoscopic Score for ulcerative colitis [23].

We did not find any association between IBD and location. However, Wędrychowicz et al. found an increased concentration of plasma elafin in children with Crohn’s disease with colonic involvement compared with patients with other locations of the disease [41]. Interestingly, Wang et al. revealed that an elevated level of elafin in the serum of patients with Crohn’s disease was significantly associated with an increased risk of intestinal strictures [23]. On the other hand, colonic elafin mRNA and protein expression were significantly lower in patients with stricturing Crohn’s disease compared with nonstricturing patients [23]. Wędrychowicz et al. found that the concentration of elafin in plasma was higher in children with inflammatory Crohn’s disease than in those with structuring or penetrating Crohn’s disease [41].

It is also important to note that Motta et al. delivered elafin in food-grade strains of lactic acid bacteria to the site of inflammation in the colon in a mouse model, which resulted in decreased elastolytic activity and inflammatory indicators [21]. Although these results have promising potential outcomes, they need to be interpreted with caution in terms of safety because elafin is overexpressed in some neoplasms, including colorectal cancer [42], high-grade serous ovarian carcinoma, and basal-like breast cancer [43].

## 5. Conclusions

To conclude, we found that serum elafin is elevated in children with IBD. Moreover, serum elafin is increased in the active phase of both ulcerative colitis and Crohn’s disease and only in the remission of ulcerative colitis. Elafin appears to be a potential candidate for a biomarker of ulcerative colitis.

## Figures and Tables

**Table 1:** Comparison of serum elafin in IBD patients in terms of disease activity to the control group.

Parameter	Groups	Mean ± SD	Median	Range	Statistical Analysis
Elafin [ng/mL]	Crohn’s disease: active phase	3.890 ± 1.655	4.448	0.139–5.870	*†‡¥§
	Crohn’s disease: remission	3.497 ± 0.737	3.479	2.484–5.088	
	Ulcerative colitis: active phase	4.795 ± 1.188	4.537	2.845–7.866	
	Ulcerative colitis: remission	4.054 ± 1.536	4.374	0.096–5.795	
	Control group	3.029 ± 1.366	3.306	0.164–4.790	

* Z = 2.18; *p* = 0.03 comparing active Crohn’s disease with control; † Z = −2.85; *p* = 0.004 comparing remissive Crohn’s disease with active ulcerative colitis; ‡ Z = −2.24; *p* = 0.03 comparing remissive Crohn’s disease with remissive ulcerative colitis; ¥ Z = −3.88; *p* = 0.0001 comparing active ulcerative colitis with controls; § Z = −2.85; *p* = 0.004 comparing remissive ulcerative colitis with controls.

**Table 2:** Diagnostic performance of serum elafin to distinguish IBD from controls.

Parameter	Cut-off	Sensitivity	Specificity	Accuracy	Positive Predictive Value	Negative Predictive Value
Elafin to distinguish ulcerative colitis from controls	4.041 ng/mL	0.721	0.850	0.762	0.912	0.586
Elafin to distinguish Crohn’s disease from controls	3.441 ng/mL	0.760	0.600	0.689	0.704	0.667

## Data Availability

The datasets generated during and/or analyzed during the current study are available from the corresponding author on reasonable request.

## References

[1] Ye Y., Manne S., Treem W.R., Bennett D. (2020). Prevalence of Inflammatory Bowel Disease in Pediatric and Adult Populations: Recent Estimates from Large National Databases in the United States, 2007–2016. Inflamm. Bowel Dis..

[2] Duricova D., Burisch J., Jess T., Gower-Rousseau C., Lakatos P.L., ECCO-EpiCom (2014). Age-related differences in presentation and course of inflammatory bowel disease: An update on the population-based literature. J. Crohns. Colitis.

[3] Collaborators G.I.B.D. (2020). The global, regional, and national burden of inflammatory bowel disease in 195 countries and territories, 1990–2017: A systematic analysis for the Global Burden of Disease Study 2017. Lancet Gastroenterol. Hepatol..

[4] Mackner L.M., Greenley R.N., Szigethy E., Herzer M., Deer K., Hommel K.A. (2013). Psychosocial issues in pediatric inflammatory bowel disease: Report of the North American Society for Pediatric Gastroenterology, Hepatology, and Nutrition. J. Pediatr. Gastroenterol. Nutr..

[5] Mackner L.M., Sisson D.P., Crandall W.V. (2004). Review: Psychosocial issues in pediatric inflammatory bowel disease. J. Pediatr. Psychol..

[6] Luther J., Dave M. (2020). Rising Inflammatory Bowel Disease Prevalence Highlights the Need for Effective, Cost-Effective Therapies. Inflamm. Bowel Dis..

[7] Kim D.H., Cheon J.H. (2017). Pathogenesis of Inflammatory Bowel Disease and Recent Advances in Biologic Therapies. Immune Netw..

[8] Prasad S.V., Fiedoruk K., Daniluk T., Piktel E., Bucki R. (2019). Expression and Function of Host Defense Peptides at Inflammation Sites. Int. J. Mol. Sci..

[9] Lei J., Sun L., Huang S., Zhu C., Li P., He J., Mackey V., Coy D.H., He Q. (2019). The antimicrobial peptides and their potential clinical applications. Am. J. Transl. Res..

[10] van der Does A.M., Hiemstra P.S., Mookherjee N. (2019). Antimicrobial Host Defence Peptides: Immunomodulatory Functions and Translational Prospects. Adv. Exp. Med. Biol..

[11] Wehkamp J., Schmid M., Stange E.F. (2007). Defensins and other antimicrobial peptides in inflammatory bowel disease. Curr. Opin. Gastroenterol..

[12] Shaw L., Wiedow O. (2011). Therapeutic potential of human elafin. Biochem. Soc. Trans..

[13] Williams S.E., Brown T.I., Roghanian A., Sallenave J.M. (2006). SLPI and elafin: One glove, many fingers. Clin. Sci..

[14] Verrier T., Solhonne B., Sallenave J.M., Garcia-Verdugo I. (2012). The WAP protein Trappin-2/Elafin: A handyman in the regulation of inflammatory and immune responses. Int. J. Biochem. Cell Biol..

[15] Wiedow O., Schroder J.M., Gregory H., Young J.A., Christophers E. (1990). Elafin: An elastase-specific inhibitor of human skin. Purification, characterization, and complete amino acid sequence. J. Biol. Chem..

[16] Elgharib I., Khashaba S.A., Elsaid H.H., Sharaf M.M. (2019). Serum elafin as a potential inflammatory marker in psoriasis. Int. J. Dermatol..

[17] Chun H.J., Yu P.B. (2015). Elafin in pulmonary arterial hypertension. Beyond targeting elastases. Am. J. Respir. Crit. Care Med..

[18] Olewicz-Gawlik A., Trzybulska D., Graniczna K., Kuznar-Kaminska B., Katulska K., Batura-Gabryel H., Frydrychowicz M., Danczak-Pazdrowska A., Mozer-Lisewska I. (2017). Serum alarm antiproteases in systemic sclerosis patients. Hum. Immunol..

[19] Flach C.F., Eriksson A., Jennische E., Lange S., Gunnerek C., Lonnroth I. (2006). Detection of elafin as a candidate biomarker for ulcerative colitis by whole-genome microarray screening. Inflamm. Bowel Dis..

[20] Schmid M., Fellermann K., Fritz P., Wiedow O., Stange E.F., Wehkamp J. (2007). Attenuated induction of epithelial and leukocyte serine antiproteases elafin and secretory leukocyte protease inhibitor in Crohn’s disease. J. Leukoc. Biol..

[21] Motta J.P., Bermúdez-Humarán L.G., Deraison C., Martin L., Rolland C., Rousset P., Boue J., Dietrich G., Chapman K., Kharrat P. (2012). Food-grade bacteria expressing elafin protect against inflammation and restore colon homeostasis. Sci. Transl. Med..

[22] Zhang W., Teng G., Wu T., Tian Y., Wang H. (2017). Expression and Clinical Significance of Elafin in Inflammatory Bowel Disease. Inflamm. Bowel Dis..

[23] Wang J., Ortiz C., Fontenot L., Xie Y., Ho W., Mattai S.A., Shih D.Q., Koon H.W. (2020). High circulating elafin levels are associated with Crohn’s disease-associated intestinal strictures. PLoS ONE.

[24] Krawiec P., Pac-Kożuchowska E. (2021). Cathelicidin—A Novel Potential Marker of Pediatric Inflammatory Bowel Disease. J. Inflamm. Res..

[25] Krawiec P., Pac-Kożuchowska E. (2020). Serum interleukin 17A and interleukin 17F in children with inflammatory bowel disease. Sci. Rep..

[26] Levine A., Koletzko S., Turner D., Escher J.C., Cucchiara S., de Ridder L., Kolho K.L., Veres G., Russell R.K., Paerregaard A. (2014). ESPGHAN revised porto criteria for the diagnosis of inflammatory bowel disease in children and adolescents. J. Pediatr. Gastroenterol. Nutr..

[27] Levine A., Griffiths A., Markowitz J., Wilson D.C., Turner D., Russell R.K., Fell J., Ruemmele F.M., Walters T., Sherlock M. (2011). Pediatric modification of the Montreal classification for inflammatory bowel disease: The Paris classification. Inflamm. Bowel Dis..

[28] Hyams J.S., Ferry G.D., Mandel F.S., Gryboski J.D., Kibort P.M., Kirschner B.S., Griffiths A.M., Katz A.J., Grand R.J., Boyle J.T. (1991). Development and validation of a pediatric Crohn’s disease activity index. J. Pediatr. Gastroenterol. Nutr..

[29] Turner D., Otley A.R., Mack D., Hyams J., de Bruijne J., Uusoue K., Walters T.D., Zachos M., Mamula P., Beaton D.E. (2007). Development, validation, and evaluation of a pediatric ulcerative colitis activity index: A prospective multicenter study. Gastroenterology.

[30] Hyams J.S., Di Lorenzo C., Saps M., Shulman R.J., Staiano A., van Tilburg M. (2016). Functional Disorders: Children and Adolescents. Gastroenterology.

[31] Motta J.-P., Martin L., Vergnolle N. (2011). Proteases/Antiproteases in Inflammatory Bowel Diseases. Proteases and Their Receptors in Inflammation.

[32] Motta J.P., Magne L., Descamps D., Rolland C., Squarzoni-Dale C., Rousset P., Martin L., Cenac N., Balloy V., Huerre M. (2011). Modifying the protease, antiprotease pattern by elafin overexpression protects mice from colitis. Gastroenterology.

[33] Henriksen P.A., Hitt M., Xing Z., Wang J., Haslett C., Riemersma R.A., Webb D.J., Kotelevtsev Y.V., Sallenave J.M. (2004). Adenoviral gene delivery of elafin and secretory leukocyte protease inhibitor attenuates NF-kappa B-dependent inflammatory responses of human endothelial cells and macrophages to atherogenic stimuli. J. Immunol..

[34] Fitch P.M., Roghanian A., Howie S.E., Sallenave J.M. (2006). Human neutrophil elastase inhibitors in innate and adaptive immunity. Biochem. Soc. Trans..

[35] Henriksen P.A., Devitt A., Kotelevtsev Y., Sallenave J.M. (2004). Gene delivery of the elastase inhibitor elafin protects macrophages from neutrophil elastase-mediated impairment of apoptotic cell recognition. FEBS Lett..

[36] Simpson A.J., Cunningham G.A., Porteous D.J., Haslett C., Sallenave J.M. (2001). Regulation of adenovirus-mediated elafin transgene expression by bacterial lipopolysaccharide. Hum. Gene Ther..

[37] McMichael J.W., Roghanian A., Jiang L., Ramage R., Sallenave J.M. (2005). The antimicrobial antiproteinase elafin binds to lipopolysaccharide and modulates macrophage responses. Am. J. Respir. Cell Mol. Biol..

[38] Sallenave J.M., Cunningham G.A., James R.M., McLachlan G., Haslett C. (2003). Regulation of pulmonary and systemic bacterial lipopolysaccharide responses in transgenic mice expressing human elafin. Infect. Immun..

[39] Roghanian A., Williams S.E., Sheldrake T.A., Brown T.I., Oberheim K., Xing Z., Howie S.E., Sallenave J.M. (2006). The antimicrobial/elastase inhibitor elafin regulates lung dendritic cells and adaptive immunity. Am. J. Respir. Cell Mol. Biol..

[40] Greuter T., Manser C., Pittet V., Vavricka S.R., Biedermann L., on behalf of Swiss IBDnet, an official working group of the Swiss Society of Gastroenterology (2020). Gender Differences in Inflammatory Bowel Disease. Digestion.

[41] Wędrychowicz A., Tomasik P., Kowalska-Duplaga K., Pieczarkowski S., Fyderek K. (2021). Plasma elafin, cathelicidin, and α-defensins are increased in paediatric inflammatory Crohn’s disease and reflect disease location. Arch. Med. Sci..

[42] Liu Y., Tian Y., Wu T., Dai Y., Wang W., Teng G. (2019). High Expression and Clinical Significance of Elafin in Colorectal Cancer. Gastroenterol. Res. Pract..

[43] Labidi-Galy S.I., Clauss A., Ng V., Duraisamy S., Elias K.M., Piao H.Y., Bilal E., Davidowitz R.A., Lu Y., Badalian-Very G. (2015). Elafin drives poor outcome in high-grade serous ovarian cancers and basal-like breast tumors. Oncogene.

